# *In vitro* Antioxidant, Cytotoxic and Antidiabetic Activities of Protein Hydrolysates Prepared from Chinese Pond Turtle (*Chinemys reevesii*)

**DOI:** 10.17113/ftb.59.03.21.7087

**Published:** 2021-09

**Authors:** Md. Serajul Islam, Wang Hongxin, Habtamu Admassu, Amer Ali Mahdi, Ma Chaoyang, Fu An Wei

**Affiliations:** 1 State Key Laboratory of Food Science and Technology, Jiangnan University, 1800 Lihu Avenue, 214122 Wuxi, Jiangsu Province, PR China; 2 National Engineering Research Center for Functional Food, Jiangnan University, 1800 Lihu Avenue, 214122 Wuxi, Jiangsu Province, PR China; 3 Biotechnology and Bioprocessing Center of Excellence, Department of Food Process Engineering, College of Biological and Chemical Engineering, Addis Ababa Science and Technology University, Addis Ababa, Ethiopia; 4 Guangxi Zhongtaikang Technology Industry Co., Ltd., 530029 Nanning, Guangxi, PRChina

**Keywords:** Chinese pond turtle, molecular mass profiles, antioxidant activity, antidiabetic capacity, lipid peroxide inhibition, cytotoxic effect

## Abstract

**Research background:**

Cardiovascular diseases and diabetes are the biggest causes of death globally. Bioactive peptides derived from many food proteins using enzymatic proteolysis and food processing have a positive impact on the prevention of these diseases. The bioactivity of Chinese pond turtle muscle proteins and their enzymatic hydrolysates has not received much attention, thus this study aims to investigate their antioxidant, antidiabetic and cytotoxic activities.

**Experimental approach:**

Chinese pond turtle muscles were hydrolysed using four proteolytic enzymes (Alcalase, Flavourzyme, trypsin and bromelain) and the degrees of hydrolysis were measured. High-performance liquid chromatography (HPLC) was conducted to explore the amino acid profiles and molecular mass distribution of the hydrolysates. The antioxidant activities were evaluated using various *in vitro* tests, including 1,1-diphenyl-2-picrylhydrazyl (DPPH) and 2,2’-azino-bis(3-ethylbenzothiazoline-6-sulfonic acid) (ABTS), hydroxyl radical scavenging activity, reducing capacity, chelating Fe^2+^ and lipid peroxide inhibition activity. Antidiabetic activity was evaluated using α-amylase inhibition and α-glucosidase inhibition assays. Besides, cytotoxic effect of hydrolysates on human colon cancer (HT-29) cells was assessed.

**Results and conclusions:**

The amino acid composition of the hydrolysates revealed higher mass fractions of glutamic, aspartic, lysine, hydroxyproline and hydrophobic amino acids. Significantly highest inhibition of lipid peroxidation was achieved when hydrolysate obtained with Alcalase was used. Protein hydrolysate produced with Flavourzyme had the highest radical scavenging activity measured by DPPH (68.32%), ABTS (74.12%) and FRAP (*A*_700 nm_=0.300) assays, α-glucosidase (61.80%) inhibition and cytotoxic effect (82.26%) on HT-29 cell line at 550 µg/mL. Hydrolysates obtained with trypsin and bromelain had significantly highest (p<0.05) hydroxyl radical scavenging (92.70%) and Fe^2+^ metal chelating (63.29%) activities, respectively. The highest α-amylase (76.89%) inhibition was recorded when using hydrolysates obtained with bromelain and Flavourzyme.

**Novelty and scientific contribution:**

Enzymatic hydrolysates of Chinese pond turtle muscle protein had high antioxidant, cytotoxic and antidiabetic activities. The findings of this study indicated that the bioactive hydrolysates or peptides from Chinese pond turtle muscle protein can be potential ingredients in pharmaceuticals and functional food formulations.

## INTRODUCTION

Cardiovascular diseases, chronic obstructive pulmonary disease (COPD), diabetes, rheumatoid arthritis and cancer are the biggest causes of death globally (*1*). Recently, food-derived bioactive peptides with therapeutic abilities have gained an increasing interest. Peptides with specific amino acid sequences that are potent in delaying and retaining the onset of diet-related diseases have been given particular attention (*2*). Food-derived protein hydrolysates or peptides as natural food resources play an important role in preventing such diseases through inhibition of α-glucosidase and α-amylase, or through antihypertensive, antioxidant, antiproliferative and antimicrobial activities (*2*, *3*). Enzymatic hydrolysis of proteins is one of the most effective approaches that can be used to release such bioactive protein hydrolysates or peptides, without affecting their nutritive value. Enzymatic protein hydrolysates contain smaller peptides derived from the larger polypeptides due to enzymatic action with 2 to 20 amino acid residues (*2*, *3*).

Lipid peroxidation in food and food products causes rancidity and results in foul taste, aroma and texture as well as reduced shelf life. Naqash and Nazeer (*4*) elucidated that severe diseases, *viz*. diabetes mellitus, neurological disorders, cardiovascular diseases and Alzheimer’s disease may occur by consuming food with oxidants. There are artificial antioxidants such as butylated hydroxyanisole, butylated hydroxytoluene, propyl gallate and *tert*-butylhydroquinone that are used to prevent lipid peroxidation in food products under strict regulation because of their health hazards (*5*). For this reason, nowadays there is a growing interest in separation and identification of antioxidant agents from natural resources that can prevent the harmful effects of reactive oxygen species (ROS) (*6*) including dietary proteins due to their potential health benefits as compared to artificial antioxidants. The bioactivity of peptides is clearly linked to smaller molecular mass, easy absorption, high activity and lack of negative side effects (*7*). Lipid peroxidation inhibition and prevention of free radical formation in food are important for protecting it from deterioration (*8*).

Diabetes mellitus is a metabolic disorder which is alarmingly increasing in the world. Ramadhan *et al.* (*9*) reported that type 2 diabetes is increasing at a rate of about 90 to 95% of cases and predicted to reach 366 million by 2030. Therefore, it is very crucial to minimize its outbreak. The most beneficial therapy for type 2 diabetes is achieved by maintaining the optimal blood glucose level after meal. Consequently, α-glucosidase and α-amylase inhibitors are important agents, because α-amylase can break down long chain carbohydrates, whereas α-glucosidase can cleave glucose from disaccharides. As a result, inhibiting these enzymes is effective in delaying glucose absorption. Obesity, free fatty acid peroxidation and a variety of oxygen-free radicals are related to diabetes. Antioxidants can scavenge the peroxides in the body and improve its antioxidant and immune capability, which helps in the prevention and treatment of diabetes mellitus (*10*). Many research studies have shown that antidiabetic peptides from animal sources contribute to type 2 diabetes prevention (*9*). On the other hand, cancer is another major cause of death in both women and men in the world (*5*). Yaghoubzadeh *et al.* (*11*) reported that antioxidants are potentially used to prevent and treat diseases associated with reactive oxygen species, including cancer. Additionally, some bioactive peptides can directly kill cancer cells or induce cell apoptosis (*12*). Although further research is required for the development of effective and less toxic drugs, there is a growing interest in the isolation and characterization of natural antitumour agents in food sources in the pharmaceutical industries.

In general, current studies are focused on the practical utilization of numerous aquatic species (*13*). Chinese pond turtle (*Chinemys reevesii*) is a commercially valuable and protein-rich edible aquatic species native to Hong Kong, China, Taiwan, Japan and Korea. It has been utilized as an ingredient for the traditional Chinese medicines (*13*). Nowadays, it is highly demanded and commercially cultivated in the above-mentioned countries. The global production of softshell turtles is estimated to 355 000 tonnes in 2014. It has been reported that Chinese softshell turtle (*Pelodiscus sinensis*), an aquatic and delicious species, has high nutritional value and is especially rich in protein and low in fat with excellent medicinal values including antioxidant, antidiabetic, anticancer as well as blood pressure-decreasing properties (*14*, *15*). Moreover, genomes of the green sea turtle (*Chelonia mydas*), Chinese softshell turtle and the Western painted turtle (*Chrysemys picta bellii*) have been investigated for their biological and nutritional properties (*16*). Therefore, in this study, Chinese pond turtle proteins were extracted, characterized and their hydrolysates were evaluated for their biological contribution to food product quality and health benefits. To the best of our knowledge, very limited or scant literature has been reported on the specificity of protein properties and production of Chinese pond turtle muscle protein hydrolysates using four proteases (Alcalase, Flavourzyme, trypsin and bromelain) and their antioxidant and antidiabetic activities, along with cytotoxic effect on human colon cancer cells. Thus, this study aims to optimize the production of Chinese pond turtle muscle protein hydrolysates by protease hydrolysis and evaluate their nutritional value, antioxidant and antidiabetic activities, and cytotoxic effect on human colon cancer (HT-29) cell line depending on different treatments.

## MATERIALS AND METHODS

### Experimental sample

Chinese pond turtle (*Chinemys reevesii*) is a kind of typical aquatic food in China. For this experiment, Chinese pond turtle was obtained from the breeding company of Guangxi Zhongtaikang Technology Industry Co., Ltd., Nanning, Guangxi, PR China. The Chinese pond turtles were euthanised immediately after arrival to the laboratory and then washed with clean water. Samples were put in the fresh ice bag before transfer to Nutrition and Function Factors Food Research Center laboratory (Jiangnan University, Wuxi, PR China). The muscles were separated manually. Finally, the selected part was minced, homogenised, packed into vacuum plastic bags and stored at -20 °C for further experiment.

### Chemicals

Alcalase 2.4L (2.4 AU-A/g) from *Bacillus licheniformis* was procured from Nanjing Chengna Chemical Company Limited (Nanjing, PR China), bromelain (300 U/mg) from pineapple, Flavourzyme (20 U/mg) from *Aspergillus oryzae*, trypsin (250 USP U/mg) from bovine pancreas, α-amylase (50 U/mg) from *Bacilus subtilis*, α-glucosidase (50 U/mg) from *Saccharomyces cerevisiae*, *p*-nitrophenyl-α-d-glucopyranoside (pNPG, ≥99%), 2,6-di-*tert*-butyl-4-methylphenol (BHT, ≥99%), acarbose (≥98%), RPMI 1640 medium and 10% fetal bovine serum (FBS) were procured from Shanghai Yuanye Biotechnology Company Limited (Shanghai, PR China). HT-29 cell was purchased from the Cell Bank of Type Culture Collection of Chinese Academy of Sciences (Shanghai, PR China). All other used reagents were of high purity and analytical grade.

### Preparation of protein hydrolysates

Protein hydrolysates were prepared as described by Noman *et al.* (*17*) with some modifications. Chinese pond turtle muscle was hydrolysed using four selected proteases under their optimal conditions as mentioned in Table 1. The pH was set by using 0.025 M sodium phosphate buffer (pH=6 to 7), and Tris-HCl buffer (pH=7.5 to 9). The enzyme activity was stopped by heating the mixture at 90 °C for 20 min using a thermostatic water bath (model HH-4; Wincom Company Ltd., Changsha, Hunan, PR China); then, the mixture was immediately transferred to an ice bath to cool, centrifuged (ST 40R; Thermo Electron LED GmbH, Langenselbold, Germany) at 8000×*g* and 4 °C for 20 min. Finally, the supernatant was collected and lyophilized under vacuum at −48 °C (SCIENTZ-10N; Ningbo Scientz Biotechnology Company Limited, Zhejiang, PR China) and the protein hydrolysates were stored at −20 °C for further experiment.

**Table 1:** The hydrolysis conditions for the preparation of protein hydrolysates from Chinese pond turtle muscle

Hydrolysis condition	Protease			
Alcalase	Flavourzyme	Trypsin	Bromelain
Incubation temperature/°C	55	50	60	55
pH	8.0	7.5	8.0	7.0
(*m*(enzyme)/*m*(substrate))/%	1, 2, 3, 4, 5	1, 2, 3, 4, 5	1, 2, 3, 4, 5	1, 2, 3, 4, 5
(*m*(solid)/*V*(liquid))/(g/mL)	1:1	1:1	1:1	1:1
*t*(incubation)/h	1, 3, 5, 7	1, 3, 5, 7	1, 3, 5, 7	1, 3, 5, 7
Inactivation temperature/°C	90	90	90	90
*t*(inactivation)/min	20	20	20	20

### Analysis of the degree of hydrolysis

Degree of hydrolysis (DH) was determined by titration as described by Noman *et al.* (*17*) with slight modification. Concisely, 1.5 g of protein hydrolysates was taken and the mass was made up to 50 g with ultrapure water. After that, the mixture was adjusted to pH=7.0 with sodium hydroxide solution (0.1 M), and then 10 mL of 38% formaldehyde solution were added and kept at 25 °C for 5 min. The solution was titrated to the end point at pH=8.5 using standard sodium hydroxide (0.1 M) solution and the consumed volume was used to calculate the amount of free amino groups (FAG). Total nitrogen (TN) in the sample was examined using the Kjeldahl method by following standard procedure (*18*). Finally, DH was calculated as follows:


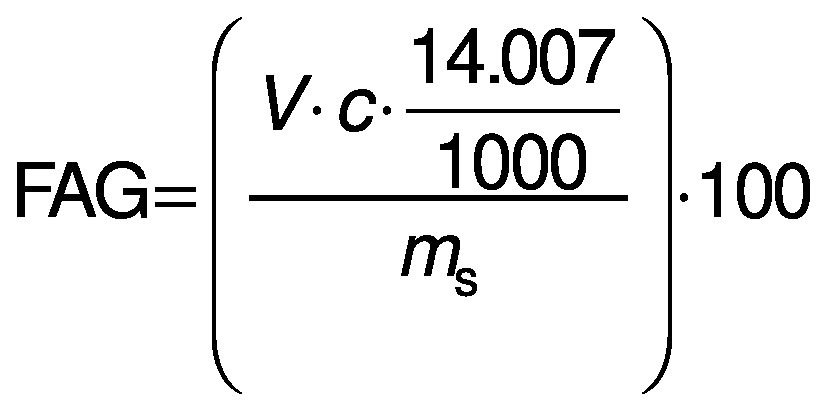



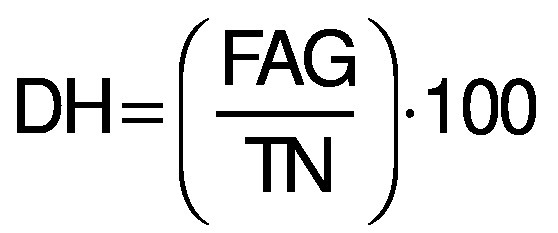


where *V* is the volume of the used 0.1 M NaOH in mL, *c* is the concentration of NaOH (0.1 M) used for titration, and *m*_S_ is the mass of the sample (g).

### Average yield and proximate composition

Average yields of the Chinese pond turtle muscle protein hydrolysates were measured according to the protocol described by Noman *et al.* (*19*) and calculated with the following formula:


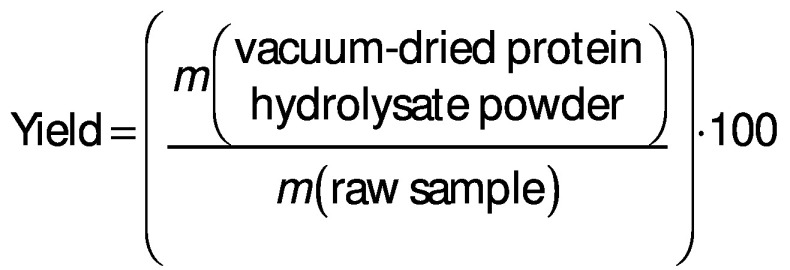


Proximate composition (moisture, protein, fat and ash contents) of the protein hydrolysates was determined using AOAC standard guideline (*18*). Briefly, moisture mass fraction was analysed by oven air drying method at 105 °C until a constant mass was obtained. Total nitrogen content was estimated by a standard micro-Kjeldahl method. Then, crude protein was calculated by multiplying total nitrogen with a nitrogen to protein conversion factor of 6.25. Ash mass fraction was analysed by incineration of the samples in a muffle furnace at 600 °C until a white ash was formed. The lipid content was determined by using macro-Soxhlet apparatus (SZG-101; Zhengzhou Laboao Instrument Equipment Co., Ltd., Shanghai, PR China) with petroleum ether.

### Amino acid composition analysis

Tryptophan was analysed by alkaline hydrolysis according to Umayaparvathi *et al.* (*20*) with minor modifications. Briefly, 100 mg of turtle muscle protein hydrolysate and 8 mL of 5 mol/L NaOH were mixed at 120 °C for 22 h under nitrogen gas and neutralised with 6.67 mL of 6 M HCl. On the other hand, other amino acids were analysed according to Noman *et al.* (*17*); the same amount of sample was taken and hydrolysed with 8 mL of 6 mol/L HCl under nitrogen gas and incubated in an oven at the same temperature and time, neutralised by 4.8 mL of 10 M NaOH. Finally, 1 μL of solutions was injected into the HPLC analytical column (250 mm×4.6 mm i.d., 5 μm particle size; Agilent Technologies, Palo Alto, CA, USA).

### Analysis of molecular mass distribution

Molecular mass distribution was investigated by gel permeation chromatography using Waters 1525 binary HPLC pump (Waters, Milford, MA, USA) and TSKgel G2000SWXL (300 mm×7.8 mm) column (Tosoh, Tokyo, Japan), as described by Noman *et al.* (*17*).

### Antioxidant activity

#### DPPH radical scavenging activity

The 1,1-diphenyl-2-picrylhydrazyl (DPPH) radical scavenging activity was evaluated according to the protocol of Umayaparvathi *et al.* (*20*). Concisely, 2 mL of protein hydrolysate sample (2, 4, 6, 8, 10 and 14 mg/mL) were added to 2 mL of 0.16 mM DPPH methanolic solution. The mixture was vortexed for 1 min and left to stand at room temperature for 30 min in a dark place, and the absorbance was read at 517 nm (UV-1800PC; Shanghai Mapada Instruments Co., Ltd, Shanghai, PR China). The ability to scavenge the DPPH radical was calculated using the following equation:


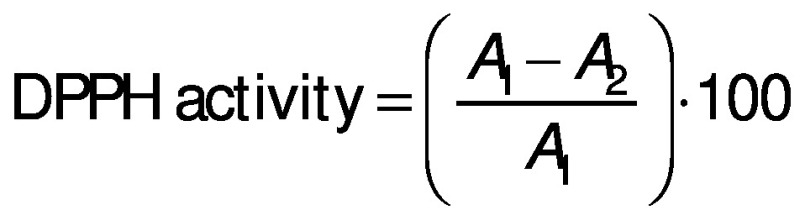


where *A*_1_ is the absorbance of the control (DPPH solution), and *A*_2_ is the absorbance of the sample (DPPH solution with sample). BHT was used as a positive control.

#### ABTS radical scavenging activity

The 2,2’-azino-bis(3-ethylbenzothiazoline-6-sulfonic acid) (ABTS) free radical scavenging assay was analysed using the method of Chi *et al.* (*12*) with slight modifications. Briefly, ABTS free radical was generated by mixing a concentration of ABTS stock solution (0.007 M potassium persulphate and 0.00245 M ABTS). The mixture was kept in the dark at room temperature for 16 h. The ABTS radical stock solution was diluted in 0.005 M phosphate-buffered saline (PBS) at pH=7.4 to an absorbance of 0.70±0.02 at 734 nm. A volume of 4 mL of diluted ABTS^•+^ solution was mixed with 0.1 mL of different concentrations of protein hydrolysates (0.5, 1, 1.5 and 2 mg/mL), and incubated at 25 °C for 10 min in a dark place. The absorbance of the mixture was measured at 734 nm (UV-1800PC; Shanghai Mapada Instruments Co., Ltd), and BHT was used as the positive control. The ABTS activity was calculated using the following equation:


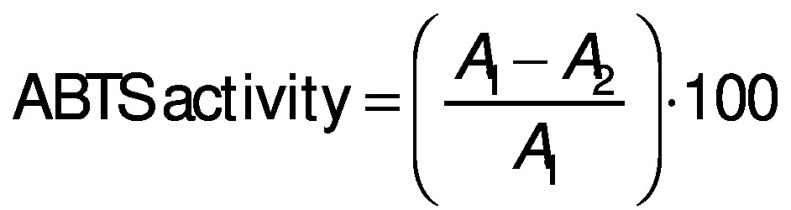


where *A*_1_ is the absorbance of control and *A*_2_ the absorbance of the sample.

#### Reducing power capacity

The ferric reducing antioxidant power (FRAP) was analysed according to the procedure by Umayaparvathi *et al.* (*20*) with slight modifications. Concisely, 2 mL of protein hydrolysates were taken at various concentrations (0.5, 1, 1.5, 2, 2.5 and 3 mg/mL) and mixed with 2 mL of phosphate buffer (200 mM, pH=6.6), and 2 mL of 1% potassium hexacyanoferrate were added. The mixture was mixed vigorously by vortex mixer (XW-80A; Ningbo Hinotek Technology Co., Ltd., Zhejiang, PR China) for 1 min and incubated at 50 °C for 25 min. Then, 1 mL of 10% trichloroacetic acid was added and mixed, then centrifuged (microcentrifuge D3024R; Scilogex, Beijing, PR China) at 10 000×*g* for 15 min. After that, the upper layer of the solution (supernatant, 2 mL) was collected and mixed with 2 mL of ultrapure water, and 0.4 mL of 0.1% FeCl_3_ was added. Finally, the mixture was incubated for 10 min at 25 °C and absorbance was measured by spectrophotometer (UV-1800PC; Shanghai Mapada Instruments Co., Ltd) at 700 nm. BHT was used as a positive control.

#### Hydroxyl radical scavenging activity

Hydroxyl radical scavenging activity of the hydrolysates was analysed according to a modified method of Chi *et al.* (*12*). In this study, sample concentration was 0.5, 1, 1.5 and 2 mg/mL. The mixtures were kept in water bath at 25 °C for 90 min and the absorbance was measured at 536 nm by a UV-1800PC spectrophotometer (Shanghai Mapada Instruments Co., Ltd.). The reaction mixture without antioxidant was used as the negative control, and a mixture without H_2_O_2_ was used as the blank. The hydroxyl radical scavenging activity (HRSA) was calculated by the following formula:


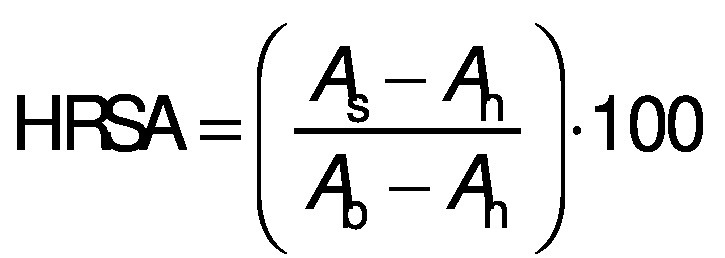


where *A*_s_, *A*_n_ and *A*_b_ are the absorbances of the sample, negative control and the blank after the reaction, respectively. BHT was used as positive control.

#### Metal chelation

The Fe^2+^ chelating ability of the hydrolysates was evaluated by the method described by Naqash and Nazeer (*4*) with minor modifications. Concisely, 3.2 mL of each sample were prepared at the concentrations of 1, 5, 10, 15 and 20 mg/mL and mixed with 40 µL of 0.002 M FeCl_2_, then the mixture was vortexed for 1 min. A volume of 80 µL of 5 mM 3-(2-pyridyl)-5,6-bis(4-phenyl-sulfonic acid)-1,2,4-triazine (ferrozine) was added to the mixture and then it was incubated at 25 °C for 15 min in a dark place. After incubation, the absorbance at 562 nm was measured with spectrophotometer (UV-1800PC; Shanghai Mapada Instruments Co., Ltd). BHT was used as a positive control. Chelating activity (%) was calculated by the following equation:


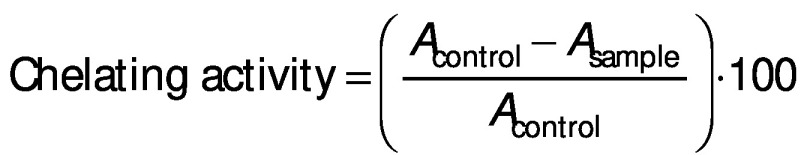


where *A*_control_ is the absorbance of the control and *A*_sample_ is the absorbance of the sample.

The IC_50_ value of the hydrolysates for antioxidant parameters such as DPPH, ABTS, ^•^OH, Fe^2+^ and BHT was determined by linear regression analysis (standard calibration curve) from a plot of concentration against the percentage of inhibition.

### Lipid peroxide inhibition assay

The lipid peroxide inhibition activity of the Chinese pond turtle muscle protein hydrolysate was analysed in a linoleic acid model system according to the method of Chi *et al.* (*12*). Concisely, the protein hydrolysates (25 mg) were dissolved in 10 mL of 0.05 M PBS (pH=7.0) and then 0.13 mL of linoleic acid and 10 mL of ethanol (99.7%) were added. Then the total volume was made up to 25 mL with ultrapure water. The mixture was incubated in a conical flask with a screw cap at (40±1) °C in the dark place, and the degree of oxidation was evaluated by measuring iron(III) trithiocyanate values. The incubated reaction solution (100 μL) was mixed with 4.7 mL of 75% CH_3_CH_2_OH, 100 μL of 30% ammonium thiocyanate (*m*/*V*) and 100 μL of 0.02 M FeCl_2_ solution in 3.5% HCl. After 3 min, the thiocyanate value was determined at 500 nm using a UV-1800PC spectrophotometer (Shanghai Mapada Instruments Co., Ltd.). BHT and α-tocopherol were used as a positive control.

### Antidiabetic activity

#### α-Amylase inhibition assay

The α-amylase assay was conducted as described by Oseguera-Toledo *et al.* (*21*) with minor modifications. The assay mixture containing 500 μL of the protein hydrolysates at different concentrations (0.1, 0.5, 1, 1.5, 2 and 2.5 mg/mL) and 500 μL of α-amylase from *B. subtilis* (1 U/mL) was pre-incubated in test tubes at 37 °C for 10 min in a water bath (model HH-4; Wincom Company Ltd). Then, 500 μL of 1% starch prepared in 0.02 mM sodium phosphate buffer at pH=6.9 with 6.7 mM NaCl were added, and the mixture was incubated for another 15 min at 37 °C. The reaction was terminated by adding 500 μL of 3,5‐dinitrosalicylic acid (DNS) colour reagent to each test tube and placed in boiling water bath for 10 min. The reaction mixture was cooled and diluted with 5 mL of ultrapure water. The absorbance was measured at 540 nm using a UV-1800PC spectrophotometer (Shanghai Mapada Instruments Co., Ltd). Control was sodium phosphate buffer (pH=6.9) and blank contained the sample and buffer without the enzyme. Acarbose (1 mg/mL) was used as a positive control. The inhibition (%) was calculated with the following formula:


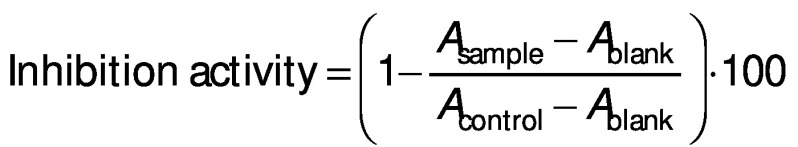


#### α-Glucosidase inhibition assay

α-Glucosidase inhibition activity was measured as described by Oseguera-Toledo *et al.* (*21*) with slight modifications. Reaction mixture containing 50 μL of protein hydrolysates at different concentrations (1, 2, 3, 4 and 5 mg/mL) and 200 μL of α-glucosidase enzyme (1 U/mL in 0.1 M phosphate buffer, pH=6.8) was preincubated at 37 °C for 15 min in a water bath (model HH-4; Wincom Company Ltd.). After incubation, 50 μL of 5 mM *p*-nitrophenyl-α-d-glucopyranoside (0.1 M phosphate buffer, pH=6.8) were added and the mixture was further incubated at 37 °C for 40 min. The reaction was terminated by the addition of 1 mL of 0.1 M Na_2_CO_3_ and the α-glucosidase activity was determined spectrophotometrically at 405 nm with UV-1800PC spectrophotometer (Shanghai Mapada Instruments Co., Ltd). Acarbose (1 mg/mL) was used as a positive control. The inhibition was calculated by the following formula:


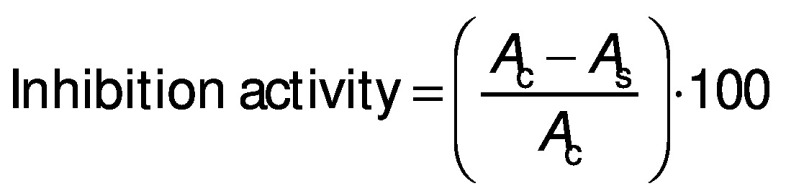


where *A*_c_ is the absorbance of the negative control and *A*_s_ is the absorbance of the sample.

The IC_50_ value of the hydrolysates for the inhibition of α-amylase and α-glucosidase activities was determined from the plot of concentration against the percentage of inhibition in a linear regression analysis.

### Analysis of cytotoxic activity by cell counting kit-8 assay

The cytotoxic activity of turtle muscle protein hydrolysates was evaluated according to the modified method of Li *et al.* (*22*). The samples were tested against human colon cancer cells (HT-29) using cell counting kit-8 (CCK-8) assay. The cells were cultured in RPMI 1640 medium with 10% feotal bovine serum (FBS) and 1% antibiotics at 37 °C in 5% CO_2_ atmosphere in 96-well microtitre plate (10·10^3^ cells per well). Stock cultures were sub-cultured two days after harvesting the cells with 0.25% trypsin-EDTA. Protein hydrolysates were dissolved in PBS (0.1 M, pH=7.4) and incubated for 24 h, then they were diluted with RPMI 1640 medium at different concentrations (100, 150, 200, 250, 300, 350, 400, 450, 500 and 550 µg/mL), placed in each well and incubated at 37 °C with 5% O_2_ for 24, 48 and 72 h, respectively. The absorbance was measured using a microplate reader (Epoch; BioTek Instruments Inc, Agilent, Winooski, VT, USA) at 450 nm. The 5-fluorouracyl (5-FU) was used as positive control. The percentage of inhibition of cytotoxic activity was measured by the following equation:


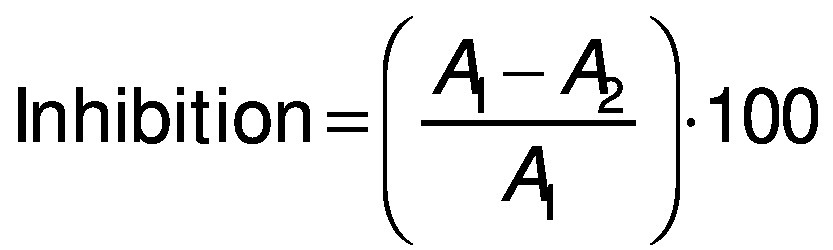


where *A*_1_ is the absorbance of the control and *A*_2_ is the absorbance of hydrolysates. The protein hydrolysate concentration which inhibits 50% of the growth was measured and recorded as IC_50_.

### Statistical analysis

All experiments were conducted in triplicate. The results were statistically analysed by one-way ANOVA using SPSS v. 22.0 software (*23*). Duncan's multiple range test was performed, the level of statistical significance was considered at p<0.05.

## RESULTS AND DISCUSSION

### Degree of hydrolysis of produced peptides

**Fig. 1:**
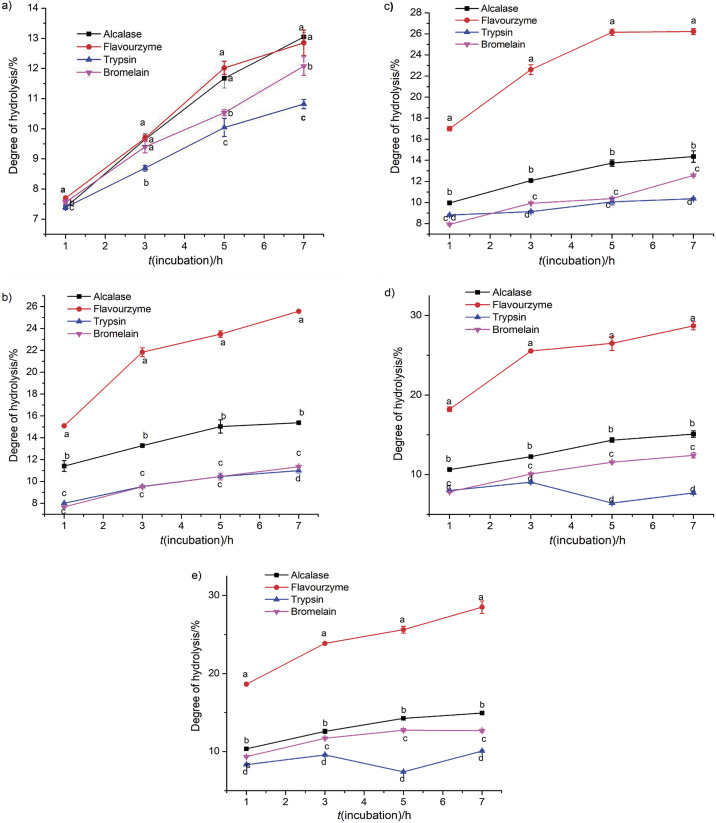
Influence of time and enzyme/substrate ratio (in %) on the degree of hydrolysis at: a) 1, b) 2, c) 3, d) 4 and e) 5%. Different lower-case letters within each assay indicate significant differences (p<0.05). Data are expressed as mean value±S.D. of triplicate measurements. S.D.=standard deviation

The four protease enzymes produced peptides with different degrees of hydrolysis (DH) under various conditions and showed significant correlation with the enzyme/substrate ratio and time (Fig. 1). As it can be observed from the results, DH increased significantly with the increase of enzyme/substrate ratio and time until the optimum conditions are reached. However, increasing the enzyme/substrate ratio and time above the optimal ratio for Alcalase and trypsin (2%, 7 h), Flavourzyme (4%, 7 h) and bromelain (5%, 5 h) did not cause any significant variation in the DH. This might be due to the enzyme aggregation, which could be caused by the inhibition of substrate diffusion, and the result is the saturation of reaction rate. In addition, the small molecular mass of peptides may be attributed to the increase in the DH.

In terms of minced Chinese pond turtle muscle, the hydrolysates produced by Flavourzyme achieved the highest DH value (28.70%) after 7 h of incubation (Fig. 1d), followed by Alcalase (15.37%) at the same incubation time (Fig. 1b), with significant (p<0.05) value. The highest DH value for bromelain was 12.75% (Fig. 1e) and for trypsin 11.01% (Fig. 1b) after 7 h of incubation. The muscle protein hydrolysates showed higher DH than the previous findings of Fu *et al.* (*24*) from bovine muscle and porcine plasma. In addition, in our study, the DH of 15.37% obtained with Alcalase was higher than reported by Karami *et al.* (*25*), where DH with Alcalase was 13.4%. Therefore, the optimal enzyme/substrate ratio and reaction time were chosen for further experiments.

### Average yield of Chinese pond turtle muscle protein hydrolysates

Average yield of freeze-dried protein hydrolysates under optimal conditions is presented in Table 2 (*26*). The yield was strongly associated with the DH, and the highest yield (22.76%) was achieved at DH=28.70% (Flavourzyme), followed by 15.03% yield at DH=15.37% (Alcalase). Furthermore, 11.93% yield was obtained with bromelain at DH=12.75 and 10.78% and DH=11.01% with trypsin. These different yields may be due to enzyme activity and temperature. Alcalase-hydrolysed protein yield was significantly higher than that reported by Galla *et al.* (*27*), who found 12.45% yield of *Labeo rohita* using Alcalase hydrolysis.

**Table 2:** Proximate composition (%), yield (%), and amino acid composition (g/100 g sample) of unhydrolysed proteins and protein hydrolysates obtained with different enzymes

Parameter	Unhydrolysed protein		Alcalase		Flavourzyme		Trypsin		Bromelain		FAO requirements*		
Child		Adult
Protein	(73.1±1.3)^c^		(80.9±0.4)^b^		(84.2±1.1)^a^		(79.7±1.8)^b^		(78.7±1.3)^b^		-		-
Moisture	(8.4±0.2)^a^		(6.7±0.1)^c^		(6.95±0.2)^bc^		(6.7±0.3)^c^		(7.2±0.3)^b^		-		-
Fat	(3.20±0.07)^a^		(0.57±0.02)^b^		(0.38±0.01)^c^		(0.55±0.02)^b^		(0.38±0.01)^c^		-		-
Ash	(8.1±0.3)^a^		(5.7±0.3)^c^		(4.5±0.4)^d^		(6.92±0.2)^b^		(6.6±0.3)^b^		-		-
Yield	-		(15.0±0.4)^b^		(22.8±0.6)^a^		(10.8±0.1^d^		(11.9±0.3)^c^		-		-
Essential amino acid													
Tryptophan		(0.31±0.01)^c^	(0.47±0.02)^b^		(0.34±0.01)^c^		(0.46±0.04)^b^		(0.55±0.03)^a^	1.1		0.5	
Histidine		(1.97±0.06)^a^	(1.93±0.05)^a^		(1.81±0.03)^b^		(1.70±0.06)^c^		(1.59±0.03)^d^	1.9		1.6	
Methionine+cysteine		(1.69±0.04)^d^	(3.0±0.1)^b^		(3.3±0.1)^a^		(2.9±0.1)^b^		(2.70±0.08)^c^	2.5		1.7	
Phenylalanine+tyrosine		(4.7±0.2)^a^	(4.1±0.3)^b^		(4.40±0.09)^ab^		(3.6±0.2)^c^		(3.6±0.2)^c^	6.3		1.9	
Threonine		(2.6±0.2)^b^	(2.21±0.05)^cd^		(2.71±0.06)^a^		(2.36±0.05)^c^		(2.10±0.03)^d^	1.4		0.9	
Isoleucine		(3.710.1)^a^	(3.1±0.1)^c^		(3.4±0.1)^b^		(3.0±0.1)^c^		(2.77±0.01)^d^	2.8		1.3	
Leucine		(5.3±0.2)^b^	(4.6±0.2)^cd^		(5.7±0.2)^a^		(4.9±0.2)^c^		(4.3±0.2)^d^	6.6		1.9	
Lysine		(5.1±0.2)^b^	(6.1±0.4)^a^		(6.2±0.2)^a^		(6.0±0.2)^a^		(5.1±0.2)^b^	5.8		1.6	
Valine		(3.8±0.1)^a^	(3.01±0.08)^c^		(3.47±0.05)^b^		(3.0±0.1)^c^		(2.81±0.07)^d^	3.5		1.3	
Non-essential amino acid													
Taurine		(0.26±0.01)^d^		(0.36±0.01)^c^	(0.45±0.02)^b^	(0.50±0.04)^a^		(0.52±0.00)^a^		-		-	
Aspartic acid		(6.2±0.2)^c^		(6.8±0.2)^b^	(7.6±0.2)^a^	(7.4±0.4)^a^		(6.7±0.2)^bc^		-		-	
Glutamic acid		(11.4±0.5)^b^		(12.8±0.4)^a^	(12.6±0.2)^a^	(12.3±0.3)^a^		(12.2±0.5)^a^		-		-	
Serine		(2.69±0.04b)^c^		(3.7±0.1)^a^	(2.81±0.08)^b^	(2.49±0.08)^d^		(2.58±0.09)^cd^		-		-	
Glycine		5.09±0.1)^b^		(4.9±0.1)^b^	(5.8±0.2)^a^	(6.0±0.2)^a^		(4.9±0.2)^b^		-		-	
Arginine		(4.34±0.31)^b^		(3.8±0.2)^c^	(4.9±0.1)^a^	(4.4±0.1)^b^		(3.5±0.1)^c^		-		-	
Alanine		(4.3±0.1)^c^		(5.6±0.1)^a^	(5.5±0.1)^a^	(5.4±0.2)^a^		(4.8±0.1)^b^		-		-	
Proline		(3.2±0.1)^a^		(3.19±0.08)^a^	(2.79±0.08)^b^	(2.53±0.09)^c^		(2.2±0.1)^d^		-		-	
Hydroxyproline		(1.04±0.05)^d^		(3.1±0.2)^b^	(3.2±0.1)^ab^	(3.4±0.1)^a^		(2.77±0.08)^a^		-		-	
*w*(amino acid)/%													
Hydrophobic		27.06		26.98	28.91	25.74		23.69		-		-	
Aromatic		5.04		4.54	4.74	4.09		4.14		-		-	
(+)charged		11.37		11.84	12.94	12.16		10.2		-		-	
(−)charged		17.61		19.62	20.12	19.72		18.85		-		-	

*FAO/WHO (*26*). Data are expressed as mean±S.D., *N*=3. Letters in superscript show significant difference at p<0.05.

FAO=Food and Agriculture Organization

### Proximate composition of unhydrolysed and hydrolysed proteins

The proximate composition of both unhydrolysed proteins and protein hydrolysates is given in Table 2. Turtle muscle protein hydrolysates had higher protein content (78.66–84.22%) than the unhydrolysed proteins (73.07%), but lower fat and ash content. This might be due to the enzymatic hydrolysis, which efficiently reduced the fat content, because of the dissolution of protein during hydrolysis and the centrifugation to separate insoluble and undigested substance. The fat content of the turtle muscle protein hydrolysates (0.38–0.57%) was lower than previous findings of Noman *et al.* (*19*), who found 7.92–11.74% fat in Chinese sturgeon using Alcalase 2.4L. Therefore, hydrolysis followed by centrifugation can be an effective alternative to market fat products with lower fat content to reduce coronary diseases.

### Amino acid profiles of protein hydrolysates

Hydrolysis with Alcalase, Flavourzyme and trypsin did not change significantly the content of most amino acids, but it was significantly affected with bromelain (Table 2). The total amino acid content of Alcalase, Flavourzyme, trypsin and bromelain hydrolysates were 72.66, 76.90, 72.27 and 65.63 g/100 g, respectively. The major amino acids in the protein hydrolysates were glutamic, aspartic and lysine, which ranged from 12.15–12.78, 6.70–7.55 and 5.08–6.19 g/100 g, respectively. Glutamic and aspartic acids are both important amino acids that contribute to palatability and flavour. In addition, alanine, glycine, serine and threonine contribute to the sweet taste (*28*). On the other hand, unhydrolysed Chinese pond turtle muscle proteins contained more valine (3.80%), isoleucine (3.71%) and proline (3.23%) than the hydrolysed ones. These results might be because all the proteins were not decomposed into peptides with different molecular mass distribution during the enzymatic hydrolysis resulting in a protein hydrolysates with a few amino acids in a slightly lower mass fraction than unhydrolysed proteins (*29*). However, the mass fractions of essential amino acids were higher than recommended by FAO/WHO (*26*) for children and adults, while the content of tryptophan and phenylalanine+tyrosine was slightly lower than those recommended for children (Table 2).

### Molecular mass distribution of protein hydrolysates

**Fig. 2:**
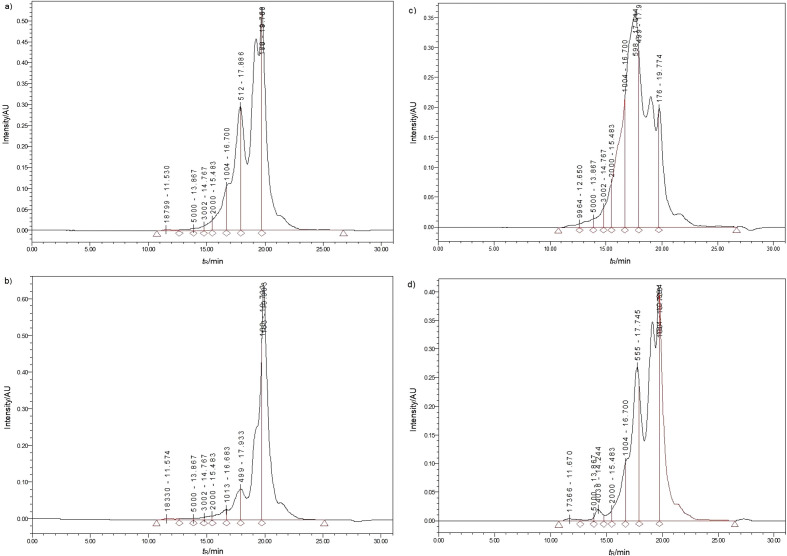
Molecular mass distribution of Chinese pond turtle muscle protein hydrolysates obtained with: a) Alcalase, b) Flavourzyme, c) trypsin, and d) bromelain. The spectra were obtained by high-performance liquid chromatography. *t*_R_=retention time

The molecular mass distribution of the hydrolysates of Chinese pond turtle muscle proteins obtained with four proteases under the optimum conditions are shown in Fig. 2. Molecular mass distribution was found to be as follows: >10 000 Da, 10 000-5000 Da, 5000-3000 Da, 3000-2000 Da, 2000-1000 Da and <1000 Da (Table S1). As clearly shown in Fig. 2, all protein hydrolysates from minced Chinese pond turtle muscle were mainly composed of low molecular mass fractions (<1000 Da), where the distributions were 95.29, 92.25, 90.75 and 78.91% for Flavourzyme, Alcalase, bromelain and trypsin hydrolysis, respectively (Table S1). Fu *et al.* (*24*) found that 60% of molecular mass of protein hydrolysates of bovine muscle and porcine plasma using ten different proteases (Alcalase, Flavourzyme, bromelain, *etc.*) was <1000 Da, which was lower than in this study. Hou *et al.* (*30*) reported the available nutritional value that remained in small molecular mass peptides (˂1000 Da), contributing to the rich dietary proteins. Additionally, it has been reported that low molecular mass peptides enhance the bioactivity such as antioxidant activity (*31*, *32*).

### Antioxidant activity of turtle muscle protein hydrolysates

#### DPPH free radical scavenging activity of protein hydrolysates

**Fig. 3:**
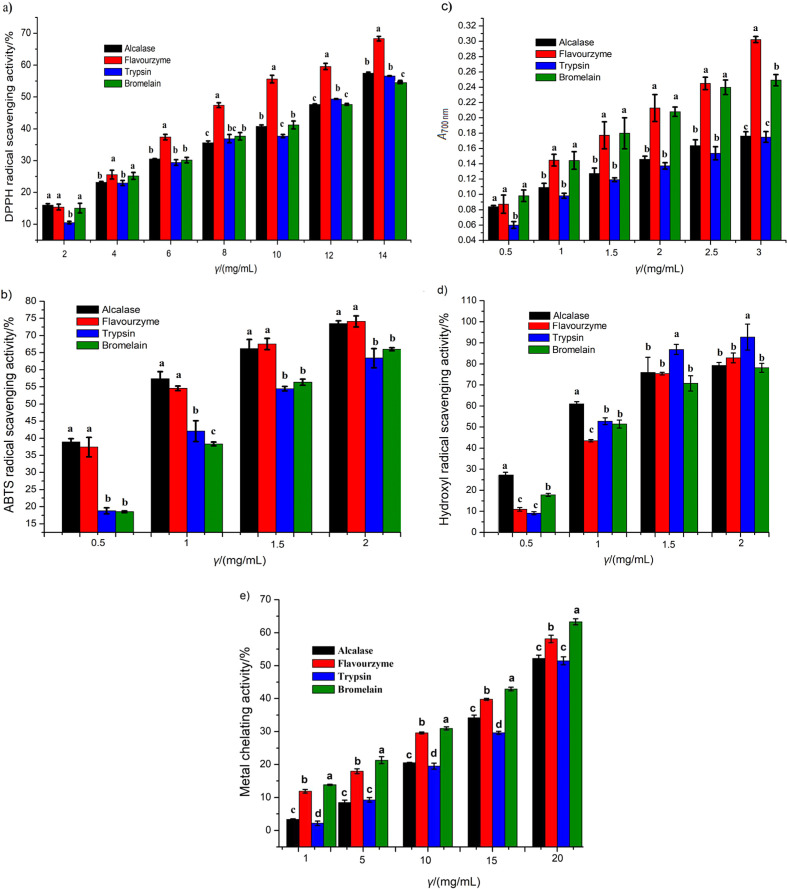
The antioxidant activities of Chinese pond turtle muscle protein hydrolysates at different concentrations: a) DPPH radical scavenging activity, b) ABTS radical scavenging activity, c) ferric ion reducing antioxidant power expressed as absorbance at *λ*=700 nm, d) hydroxyl radical scavenging activity, and e) metal (Fe^2+^) chelating activity. All results are presented as mean value±S.D. (*N*=3) of triplicate measurements. Different letters above the bars represent significant differences at p<0.05

The DPPH free radical scavenging activity of the protein hydrolysates obtained with various enzymatic treatments is shown in Fig. 3a. The results showed that Flavourzyme hydrolysate achieved the highest DPPH activity of 68.32% (14 mg/mL), followed by Alcalase (57.45%) at the same concentration. On the other hand, trypsin hydrolysate had significantly (p<0.05) higher DPPH radical inhibitory activity than bromelain. The current results of antioxidant activities of the turtle muscle protein hydrolysates may be related to amino acid composition, DH and molecular mass distribution of peptides that are electron donors and may react with free radicals to convert them to more stable products. Park *et al.* (*33*) reported that the amino acids, including threonine, valine, isoleucine and hydrophobic amino acids strongly contributed to the enhanced DPPH scavenging activity.

The IC_50_ value of Flavourzyme hydrolysate was (9.36±0.21) mg/mL, so it was stronger than the other three: Alcalase ((12.21±0.17) mg/mL), trypsin ((12.21±0.31) mg/mL) and bromelain ((12.38±0.29) mg/mL). However, DPPH inhibitory activity of turtle muscle protein hydrolysates was significantly lower than that of the standard BHT (IC_50_=(0.16±0.00) mg/mL). The percentage of DPPH inhibition was higher than of housefly larva hydrolysates obtained with Alcalase 2.4L and Flavourzyme (*34*). The current study noticeably showed that Flavourzyme hydrolysate had the highest DPPH radical scavenging activity because it contained more electrons to donate and convert the free radicals into the more stable products by terminating the radical chain reactions.

#### ABTS free radical scavenging activity of protein hydrolysates

ABTS radical assay is an excellent tool for evaluation of the antioxidant activity, in which the radical scavenging is slaked to form ABTS radical complex (*35*). As clearly shown in Fig. 3b, the highest inhibitory activity (74.12%) of ABTS^•+^ was achieved by Flavourzyme hydrolysate followed by Alcalase (73.49%) at the concentration of 2 mg/mL, with no significant difference (p≥0.05). However, bromelain (65.99%) and trypsin (63.40%) hydrolysates showed significant differences (p<0.05) of scavenging activity at the concentration of 2 mg/mL. These research results were in agreement with Chi *et al.* (*12*), who found 85.10% activity at the concentration of 2.5 mg/mL of blood clam (*Tegillarca granosa*) muscle. Hassan *et al.* (*36*) reported that some amino acids (cysteine, tryptophan, tyrosine and histidine) showed better ABTS scavenging activity. It was also found that the active peptides mainly with small molecular mass were responsible for antioxidant activity (*32*). These findings were closely related to the current research results (Table 2 and Fig. 2). The IC_50_ values of Alcalase, Flavourzyme, bromelain and trypsin hydrolysates against ABTS radicals were (0.85±0.03), (0.91±0.01), (1.41±0.03) and (1.43±0.07) mg/mL, respectively, which means they are worse antioxidants than BHT (IC_50_=(0.09±0.004) mg/mL). The results show that Alcalase and Flavourzyme hydrolysates have better ABTS radical scavenging activities than the other hydrolysates, so they could be used as potential natural antioxidants.

#### Ferric ion reducing antioxidant power of protein hydrolysates

The ferric ion reducing antioxidant power (FRAP) of turtle muscle protein hydrolysates is shown in Fig. 3c. The results clearly indicate that the reducing power of protein hydrolysates increased significantly (p<0.05) with the increase in their concentration. The highest absorbance value was recorded for Flavourzyme hydrolysate (0.30), followed by bromelain (0.25) at the concentration of 3 mg/mL, showing significant (p<0.05) difference. Alcalase and trypsin hydrolysates showed lower absorbances of 0.180 and 0.175, respectively, with no significant (p≥0.05) difference. Thus, the increase in the absorbance indicated higher reducing power of the hydrolysates. The strong capability of Flavourzyme hydrolysate may be attributed to the presence of H^+^ (protons and electrons) generated during peptide cleavages. The reducing power was higher in the Chinese pond turtle muscle protein hydrolysates than in Spanish mackerel skin hydrolysate (*37*) and haemoglobin hydrolysate (*38*). Although the values of absorbances obtained in this study were lower than the commercial BHT, which is 0.57 at the concentration of 0.1 mg/mL, these turtle protein hydrolysates can be used as a potential reducing agent. In the present study, reducing power ability was different in all enzymatic treatments, possibly due to enzyme specificity to hydrolyse the substrate and properties of the chemicals used for extraction of protein hydrolysate from Chinese pond turtle muscle. In addition, Cumby *et al.* (*39*) found significant differences in reducing capacities of the prepared hydrolysates, which might be due to the substrate specificity.

### Hydroxyl radical scavenging activity of protein hydrolysates

Hydroxyl radical is highly capable of attacking and destroying the biomolecules in living cells such as proteins, amino acids and lipids (*40*). Thus, hydroxyl radical scavenging activity evaluation is an important parameter that can provide valuable information on the antioxidant activities of peptides. The hydroxyl radical scavenging activity of Chinese pond turtle muscle protein hydrolysates is presented in Fig. 3d. Potentially significant (p<0.05) scavenging activity was observed in trypsin hydrolysate (92.70%) followed by Flavourzyme (82.85%) at concentration of 2 mg/mL, but Alcalase and bromelain hydrolysates exhibited scavenging activity without significant difference (p≥0.05) at the same concentration. The IC_50_ values for the hydroxyl radical scavenging activity were (0.69±0.02), (0.83±0.05), (0.94±0.02) and (1.14±0.07) mg/mL for trypsin, Flavourzyme, Alcalase and bromelain, respectively, which were lower than of BHT standard (IC_50_=(0.24±0.01) mg/mL). It can be seen from the results that bromelain hydrolysate exhibited higher IC_50_ value, showing a lower hydroxyl radical scavenging activity than the other enzymatic hydrolysates. In general, in this study, turtle muscle protein hydrolysates showed an excellent ^•^OH scavenging activity, so they could be used as a scavenger for reducing the damage induced by hydroxyl radicals in biosystems, food and pharmaceutical products.

### Metal (Fe^2+^) chelating activity of protein hydrolysates

Metal chelators act as catalysts that reduce the accessibility of transition metals and protect the radical-mediated oxidative chain reactions in food or biological systems. Therefore, they are necessary to improve the food quality, food safety and stability as well as human health benefits (*41*). The chelating activity of turtle muscle protein hydrolysates with different enzymatic hydrolysis is given in Fig. 3e. As it can be seen, the Fe^2+^ chelating activity increased significantly (p<0.05); the highest result achieved was 63.29% for bromelain, followed by 58.14% for Flavourzyme hydrolysate. The lowest values were 52.19 and 51.46% for Alcalase and trypsin hydrolysate, respectively. These results may be due to the incapability of small peptides to form the complex with metals. Hamzeh *et al.* (*42*) reported that metal chelating activity decreased with increasing DH. Also Noman *et al.* (*19*) found that metal ion chelating activity was ≥52% at 50 mg/mL of Chinese sturgeon hydrolysate, which was lower than of the Chinese pond turtle muscle protein hydrolysate. The IC_50_ values of the turtle muscle protein hydrolysates were obtained in the increasing order: (16.36±0.31), (17.95±0.50), (18.33±0.74) and (18.89±0.27) mg/mL for bromelain, Flavourzyme, Alcalase and trypsin hydrolysate, respectively, with significant differences between bromelain and Flavourzyme. However, no significant (p≥0.05) difference was observed between Alcalase and trypsin hydrolysates. Their IC_50_ values were lower than the positive control BHT ((3.87±0.12) mg/mL). However, the protein hydrolysates can play a potential role as a natural antioxidant source.

### Lipid peroxidation inhibition of turtle muscle protein hydrolysates

**Fig. 4:**
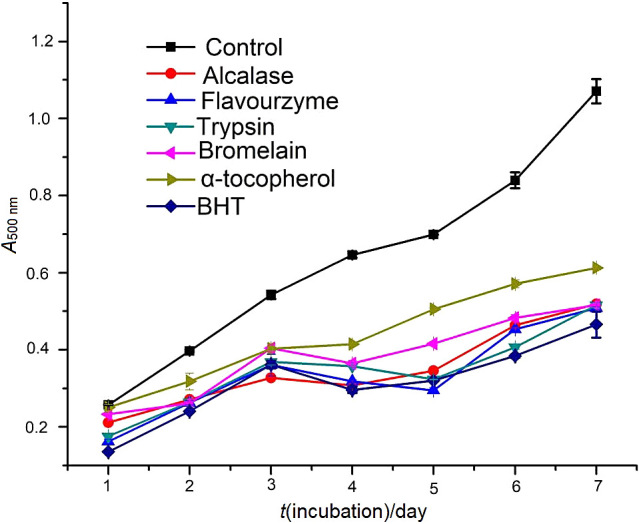
Lipid peroxidation inhibition activity of Chinese pond turtle muscle protein hydrolysates at various concentrations. All results are expressed as mean value±S.D. of triplicate measurements

Lipid oxidation is the main cause of food spoilage. Its inhibition is an important indicator for evaluating antioxidant activity of protein hydrolysates or peptides, which initiates a sequence of reactions that can generate ketones, aldehydes and other potentially toxic substances (*43*). Thus, the lipid peroxidation inhibition activities of Alcalase, Flavourzyme, trypsin and bromelain hydrolysates were evaluated at the concentration of 1 mg/mL using linoleic acid and the results achieved after seven days of incubation are given in Fig. 4. The highest absorbance value of the negative control (without antioxidant) indicated the highest level of linoleic acid hydroperoxides. Compared with the negative control, the positive control (α-tocopherol and BHT) of the four enzymatic hydrolysates strongly inhibited lipid peroxidation in the system during incubation.

The incubation period of 1 to 7 days showed that natural antioxidant α-tocopherol had significantly (p<0.05) lower inhibition activity than the four enzymatic hydrolysates, but the activity of synthetic BHT was higher than of all hydrolysates, except for the incubation for 3 days of Alcalase and Flavourzyme hydrolysates, but not of trypsin and bromelain hydrolysates. As illustrated in Fig. 4, Alcalase hydrolysate had higher activity than other hydrolysates on days 1, 5 and 7, but after 7 days without significant (p≥0.05) difference. Similarly, Flavourzyme hydrolysate had higher activity on days 3 and 4. On the 6th day of incubation, the results of the protein hydrolysis were in the following sequence: trypsin>alcalase>Flavourzyme>bromelain. The capability of bromelain hydrolysate to inhibit linoleic acid oxidation on all incubation days (except on the 2nd day) may be due to the depletion of free electrons (*6*). In addition, aromatic and/or hydrophobic amino acids (Table 2), which could lead to more interactions between peptides and fatty acids by increasing the peptide solubility in lipids, may increase the oxidation prevention (*44*). Our results support those by Chi *et al.* (*12*). However, the results of this study show that Chinese pond turtle muscle protein hydrolysates had superior inhibition of linoleic acid oxidation when compared to lipid peroxidation inhibitory activity of rapeseed protein hydrolysates at the same concentration (*6*).

### Antidiabetic activity of turtle muscle protein hydrolysates

#### *In vitro* α-amylase inhibitory activity of protein hydrolysates

**Fig. 5:**
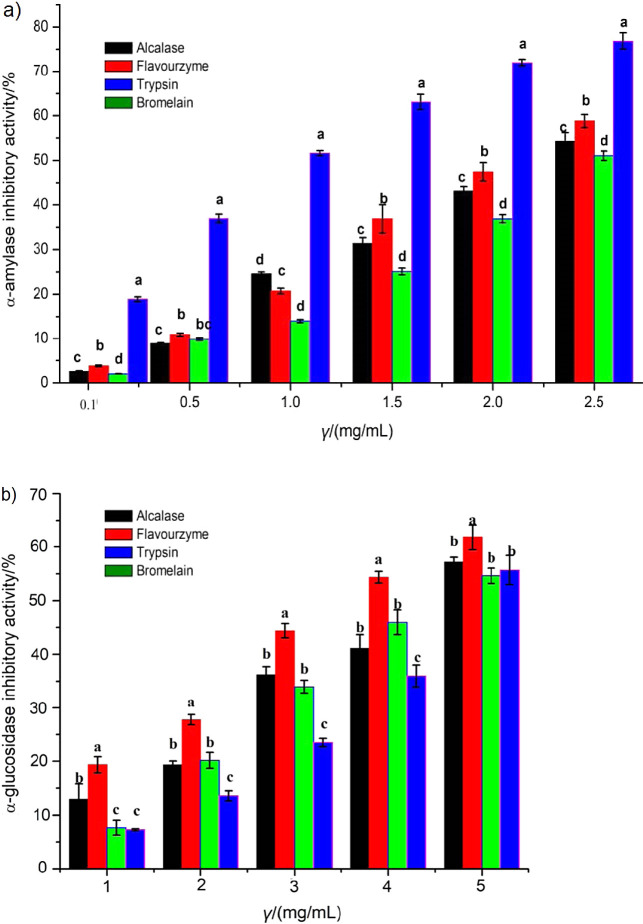
*In vitro* antidiabetic activities of Chinese pond turtle muscle protein hydrolysates using: a) α-amylase, and b) α-glucosidase and compared with Alcalase, Flavourzyme, trypsin and bromelain. Results are presented as mean value±S.D. (*N*=3). Bars at the same concentration but with different lower-case letters are significantly different at p<0.05

Alcalase, Flavourzyme, trypsin and bromelain hydrolysates expressed a significant inhibition against α-amylase enzymatic activity (Fig. 5a). The strongest obtained α-amylase inhibitory activity was 76.89% for bromelain, followed by Flavourzyme (58.79%), alcalase (54.25%) and trypsin (51.03%) hydrolysate at the concentration of 2.5 mg/mL. This may be due to the peptides having branched chain amino acids (tryptophan, phenylalanine, lysine, tyrosine and valine) and cationic residues preferably bound to α-amylase (*45*). The IC_50_ value was: (1.13±0.02), (2.12±0.05), (2.31±0.04) and (2.41±0.07) mg/mL for bromelain, Flavourzyme, Alcalase and trypsin hydrolysate, respectively. However, these values were lower than of the standard acarbose (IC_50_=(0.71±0.03) mg/mL). This may be because acarbose is an artificial purified inhibitor of α-amylase, whereas the turtle muscle protein hydrolysates are the mixtures of peptides, probably with some non-protein components present. Connolly *et al.* (*46*) wrote that type 2 diabetes management using acarabose drug was associated with negative side effects (such as abdominal dissention, meteorism, flatulence and probable diarrhoea). Thus, Chinese pond turtle muscle protein hydrolysate can play a great role as natural source of antidiabetic agent to substitute acarbose if the hydrolysates can be further purified.

#### *In vitro* α-glucosidase inhibitory activity of protein hydrolysates

The α-glucosidase inhibitors have an important role in the prevention of type 2 diabetes, which can decrease the absorption of carbohydrates and reduce the postprandial hyperglycaemia (*47*). As illustrated in Fig. 5b, the α-glucosidase inhibitory activity significantly (p<0.05) increased with increasing the concentrations of four enzymatic hydrolysates from 1 to 5 mg/mL. The IC_50_ values against α-glucosidase inhibitory activity were: (3.76±0.08), (4.46±0.15), (4.51±0.03) and (4.91±0.10) mg/mL for Flavourzyme, trypsin, Alcalase and bromelain hydrolysate, respectively, where the inhibitory activities of the hydrolysates were much lower than of acarbose (IC_50_=(1.44±0.01) mg/mL). All hydrolysates had good inhibitory activity against α-glucosidase, whereas Flavourzyme inhibitory activity was stronger than of the other hydrolysates (Alcalase>bromelain>trypsin). Yu *et al.* (*48*) found that both α-amylase and α-glucosidase inhibitors were compounds that help in the control of diabetes by decreasing the absorption of glucose.

### Cytotoxic effect of turtle muscle protein hydrolysates

**Fig. 6:**
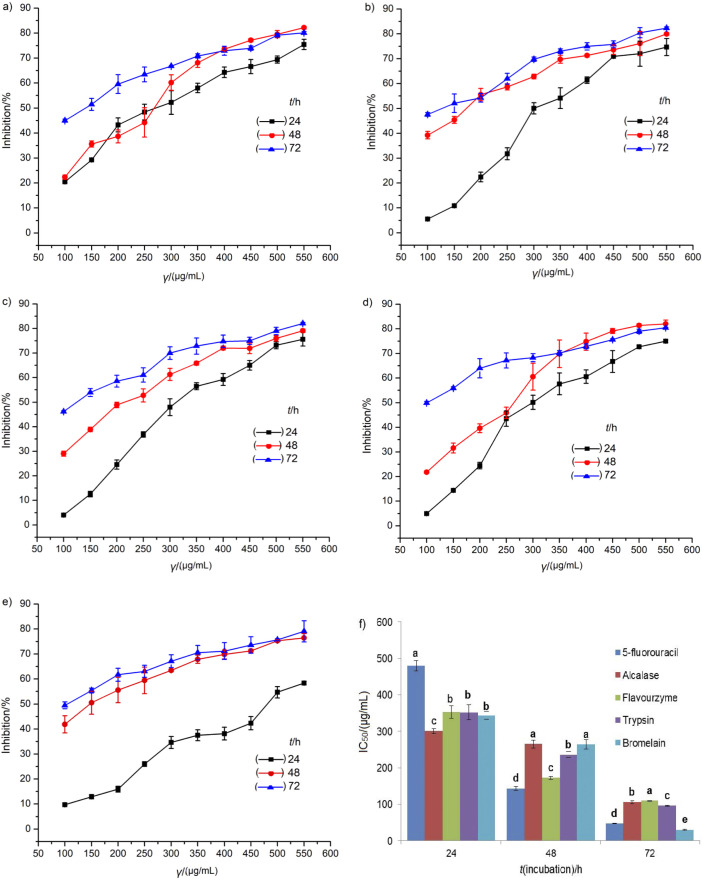
Cytotoxic effects of Chinese pond turtle muscle protein hydrolysates at concentrations of 100 to 550 µg/mL on HT-29 cell lines over a period of 24, 48 and 72 h: a) Alcalase, b) Flavourzyme, c) trypsin, d) bromelain, e) 5-fluorouracil, and f) IC_50_ values. The values are given as mean±S.D. (*N*=3). Bars with different letters differ significantly (p<0.05)

The inhibitory effect observed after 24, 48 and 72 h of incubation at the same concentration is shown in Fig. 6. As illustrated in Fig. 6b, the maximum HT-29 cell inhibition was obtained by Flavourzyme hydrolysate (82.26%) with the concentration of 550 µg/mL at 72 h of incubation. The cytotoxic effect of hydrolysates on HT-29 cells was increased with increasing incubation time. The incubation periods of 48 and 72 h gave better results than 24 h incubation period. Hence, the results indicated that the protein hydrolysates showed a perceptible dose- and time-dependent cytotoxic effect on colon cancer cells. The present findings are in agreement with the findings of Umayaparvathi *et al.* (*5*), who studied the hydrolysates of oyster (*Saccostrea cucullata*) on the cytotoxicity against HT-29 cell line during time. In this study, the cytotoxic activity of Chinese pond turtle muscle hydrolysates was higher than that determined by Wikarta and Kim (*49*), who detected cytotoxic activity of solitary tunicate hydrolysate at 1 mg/mL against stomach (AGS), human colon (DLD-1) and cervical (HeLa) cancer cells. Alemán *et al.* (*50*) also observed cytotoxic activity of squid gelatin Alcalase hydrolysate against MCF-7 cell lines (41.64%) at concentration of 1 mg/mL.

Furthermore, as observed in Fig. 6f, the IC_50_ values of the muscle protein hydrolysates were better than the commercial standard drug 5-FU after incubation for 24 h. However, Alcalase, Flavourzyme and trypsin hydrolysates showed slightly lower values after 24 and 72 h incubation than the standard drug 5-FU. Similarly, after 48 h of incubation, Flavourzyme hydrolysate had strong cytotoxic activity (IC_50_=(172.49±4.10) µg/mL), followed by trypsin (IC_50_=(236.04±8.06) µg/mL). The findings of the present study were slightly lower than the findings of Umayaparvathi *et al.* (*5*), who reported the cytotoxic activity of oyster hydrolysates on HT-29 cells with IC_50_ value of (90.31±0.45) µg/mL (except bromelain hydrolysate at 72 h). However, our experimental value was higher than that by Karami *et al.* (*25*), who obtained IC_50_=12.94 mg/mL of wheat germ protein hydrolysate prepared with Alcalase on A549 lung cancer cell line. The significant variations of Chinese pond turtle muscle protein hydrolysate cytotoxic activities may contribute to various chemical compositions of proteins originating from differences in enzyme specificity, extraction as well as the type of used cancer cells.

## CONCLUSIONS

The use of different proteolytic enzymes of Chinese pond turtle muscle proteins leads to different molecular mass distribution, degree of hydrolysis and amino acid composition of protein hydrolysates, which greatly influences their cytotoxic, antioxidant and antidiabetic activity potential. The protein hydrolysates showed antioxidant activities (DPPH, ABTS and FRAP), which were significantly higher when using Flavourzyme, whereas hydroxyl radical scavenging activity was more pronounced in trypsin hydrolysate and iron metal chelating in bromelain hydrolysate. Alcalase and Flavourzyme hydrolysates showed stronger inhibition of linoleic acid oxidation than other hydrolysates. Bromelain hydrolysates demonstrated the strongest α-amylase inhibitory activity and Flavourzyme hydrolysates showed α-glucosidase inhibitory capability. Moreover, Flavourzyme hydrolysate had the highest cytotoxic effect against colon cancer cell line (HT-29). These results suggest that Chinese pond turtle muscle hydrolysates can be used as an alternative new natural material in the development of functional foods with potential cytotoxic, antidiabetic and antioxidant activities.

## Figures and Tables

**Table S1:** Molecular mass distribution in Chinese pond turtle muscle hydrolysates obtained with four proteolytic enzymes under the optimum conditions of enzyme/substrate ratio and reaction time: Alcalase and trypsin (2%, 7 h), Flavourzyme (4%, 7 h) and bromelain (5%, 5 h)

*M*_r_/Da	*M*_r_(distribution)/%			
Alcalase	Flavourzyme	Trypsin	Bromelain
>10000	0.19	0.43	0.42	0.45
10000-5000	0.29	0.43	1.06	0.27
5000-3000	0.52	0.52	1.60	1.37
3000-2000	1.05	0.76	3.17	0.90
2000-1000	5.70	2.57	14.84	6.26
<1000	92.25	95.29	78.91	90.75
